# Recombinant proteins from *Gallibacterium anatis* induces partial protection against heterologous challenge in egg-laying hens

**DOI:** 10.1186/s13567-016-0320-6

**Published:** 2016-02-25

**Authors:** Susanne Elisabeth Pors, Ragnhild Bager Skjerning, Esben M. Flachs, Anders Miki Bojesen

**Affiliations:** Department of Veterinary Disease Biology, Faculty of Health and Medical Sciences, University of Copenhagen, Frederiksberg C, Denmark; Department of Occupational and Environmental Medicine, Bispebjerg University Hospital, Copenhagen, Denmark

## Abstract

*Gallibacterium anatis* is a Gram-negative bacterium and major cause of salpingitis and peritonitis in egg-laying hens, thereby contributing to decreased egg production and increased mortality among the hens. Due to widespread drug resistance and antigenic diversity, novel prophylactic measures are urgently required. The aim of the present study was to evaluate the cross-protective capacity of three recombinant proteins recently identified as potential vaccine candidates; GtxA-N, GtxA-C, and FlfA, in an in vivo challenge model. Nine groups of birds were immunized twice with each protein, respectively, with 14 days separation. Additionally, three groups served as non-immunized controls. After 3 weeks, the birds were challenged with either of three *G. anatis* strains: 12656-12, 7990 or IPDH 697-78, respectively. Blood samples were taken at three different time points prior to challenge, as well as 48 h after challenge. All birds were euthanized and subjected to a post mortem procedure including scoring of lesions and sampling for bacterial growth. Moreover, ELISA assays were used to quantify antigen-specific IgG titers in serum. The results showed that all three proteins induced protection against the homologous strain 12656-12. No protein induced complete protection against strain 7990, although FlfA reduced the bacterial re-isolation rate. Moreover, immunization with GtxA-N and FlfA induced protection, while GtxA-C reduced the bacterial re-isolation, against strain IPDH 697-78. Thus although complete cross-protection against all three strains was not achieved, the results hold great promise for a new generation of immunogens in the search for novel prophylactic measures against *G. anatis.*

## Introduction

Current strategies to prevent and treat bacterial salpingitis and peritonitis among egg-laying hens have not been able of effectively diminish the occurrence of the disease, which remains to be a common problem in modern poultry production. Salpingitis and peritonitis has a profound negative impact on egg production and increases the mortality among commercial layers [1–3]. Among the bacterial agents associated with salpingitis and peritonitis is the Gram-negative bacterium *Gallibacterium anatis* [4]. Even though the pathogenesis of salpingitis and peritonitis caused by *G. anatis* has not been completely elucidated [5], a number virulence factors have been reported [6]. *Gallibacterium anatis* can also be found as part of the normal microbiota of the chicken upper respiratory tract and lower genital tract [7, 8]. Unfortunately, *G. anatis* field strains are characterized by a high antigenic diversity [9, 10], and widespread antibiotic resistance has been reported [11], making current treatments inefficient on a broad scale. Consequently, there is a high demand for new innovative prevention strategies, such as a broadly-protective vaccine, to efficiently prevent infections caused by *G. anatis*.

In general, development of a successful vaccine inducing broad heterologous protection against bacteria with high antigenic diversity is particularly challenging [12]. The antigenic diversity demands the use of common antigens providing a broadly-based immunity, which can be challenging to identify. For other poultry-associated pathogens showing similar high antigen variability, e.g., *Pasteurella multocida* and *Escherichia coli*, some successful attempts have been made to identify vaccine candidates with a broad protection [13–15]. In the recent years, a pan-genomic reverse vaccinology approach has been developed to identify highly conserved antigens [16, 17] by which, Bager et al. identified a number of conserved and potential immunogens of *G. anatis* [18]. Some of these have proven to have an immunogenic potential against homologous challenge, including the recombinant forms of the N-terminal of GtxA, a large cytolytic RTX toxin with both the haemolytic and leukotoxic activity [19, 20], as well as the F17-like fimbrial protein FlfA subunit of the F17-like fimbriae [21]. These proteins are widespread among the *Gallibacterium* strains of different origin [19, 22], and therefore represent promising candidates for induction of protective immunity in a serotype-independent manner.

In the present study, we investigated the cross-protective potential of the three potential vaccine candidates; GtxA-N (N-terminal of GtxA), GtxA-C (C-terminal of GtxA) and FlfA, against three different serotypes of *G. anatis* using an in vivo challenge model.

## Materials and methods

### Strains of *Gallibacterium anatis* used for heterologous challenge

Three different strains of *G. anatis* biovar *heamolytica* were used to study the cross-protective potential; the homologous strain 12656-12 (biovar 4) isolated from liver lesions in a Danish chicken [1], as well as strain IPDH 697-78 (biovar 15), isolated from a diseased chicken in Germany [7], and strain 7990 (biovar 3), isolated from a Mexican chicken with lesions [19].

### Birds and housing

Two experiments were used in the present study: (1) Initial validation of the immunogenic potential of all three proteins with homologous challenge (strain 12656-12), using 80 Isa Brown layers, 19 weeks of age, and (2) Evaluation of protection against homologous (strain 12656-12) and heterologous challenge (strain IPDH 697-78 and 7990), using a total of 240 Isa Brown layers, 18 weeks of age. All birds were purchased from a commercial breeder, housed on the floor in groups of 20 birds with access to ad libitum feed, water and nesting material. At arrival, the birds were allowed one week of acclimatization before initiation of the experiments. Prior to immunization, all birds were swabbed in the cloaca. The swabs were cultivated on brain heart infusion (BHI) agar (Oxoid) with 5% citrated bovine blood in sealed plastic bags at 37 °C overnight to test for the presence of *G. anatis*. A total of 25 birds showed feather pecking behavior and were removed from the study between day 14 and day 35, in order to prevent development cannibalism among the birds. All experiments were approved by the Danish Animal Inspectorate (license number 2012-15-2934-00339).

### Immunization of laying hens

A total of 80 birds were immunized with either one of the following recombinant proteins; GtxA-N, GtxA-C or FlfA (immunized), or with a placebo (non-immunized; Table 1). The recombinant proteins were expressed and purified as described in Bager et al. [18]. Immunization was done using 100 µg protein in 0.5 mL of protein buffer and 0.5 mL Freund incomplete adjuvant (Sigma). For soluble proteins (GtxA-N and FlfA), soluble protein buffer (50 NaP, 150 NaCl, 0.5 mM TCEP, 10% glycerol; pH 7.5) was used, while insoluble protein buffer (100 mM sodium phosphate [pH 7.4], 0.15 M NaCl, 8 M urea) was used for the insoluble protein GtxA-C. The non-immunized group received 0.5 mL soluble protein buffer and 0.5 mL Freund incomplete adjuvant (Sigma). Two subcutaneous immunizations were performed in the neck with 2 weeks of separation.

**Table 1:** **Immunization and challenge**

Protein	Challenge strain	No. of birds (day 0/35)^a^
GtxA-C	7990	20/19
GtxA-N		20/19
FlfA		20/17
Placebo		20/17
GtxA-C	IPDH 697-78	20/20
GtxA-N		20/20
FlfA		20/20
Placebo		20/15
GtxA-C	12656-12	40/38
GtxA-N		40/39
FlfA		40/36
Placebo		40/35

Immunization with one of three recombinant proteins was done at day 0 and 14, followed by challenge with a *G. anatis* strain at day 35.

^a^ A total of 25 birds showed feather pecking behavior and were removed from the study between day 14 and day 35, in order to prevent development cannibalism among the birds.

### Challenge experiments

Three weeks after the second immunization, all groups were challenged with *G. anatis* (Table 1). All strains, kept at −80 °C, were incubated at 37 °C on BHI blood agar in a closed plastic bag. One colony of each strain was inoculated into BHI broth and incubated at 37 °C overnight. The following day, 10 mL culture was transferred to fresh BHI and incubated until reaching early exponential growth phase. Each bird received 1 mL of the inoculum intraperitoneally, corresponding to 10^8^ CFU/mL.

### Post mortem examination

After euthanasia at 48 h post challenge, gross lesions found in the peritoneum, ovary and oviduct were recorded and scored from 0 to 5 according to severity (Table 2). When analyzing the data, a binary division of the lesion scores was made according to presence or absence of a protective response (Table 2). Lesion scores 0, 1 and 2 were regarded as inflammatory reaction without significant infection and considered as a protective response against infection. Lesions scores 3, 4 and 5 were regarded as inflammation with infection and considered as a response with no protection, i.e., infection had overwhelmed the inflammatory response. Furthermore, swabs were sampled from the peritoneum, ovary and three fixed points in the oviduct; infundibulum, magnum and uterus. All swabs were streaked on BHI blood agar plates and incubated in a closed plastic bag for 18 h at 37 °C. The growth of *G. anatis* was scored from 0 to 4: 0 (no colonies), 1 (<10 colonies), 2 (10–200 colonies), 3 (>200 colonies), and 4 (dense/florid growth/distinction of colonies not possible). The total bacterial load was calculated by summation of all scores giving each bird maximal total score of 20.

**Table 2:** **Scoring of lesions in birds infected with** ***Gallibacterium anatis***

Organ	Description	Score	Protective response
Peritoneum	Normal	0	Yes
	Mild cloudiness	1	
	Moderate cloudiness and serous exudates	2	
	Moderate cloudiness and serous exudates with fibrin spots	3	No
	Complete cloudiness and local fibrinopurulent exudates	4	
	Complete cloudiness and diffuse fibrinopurulent exudate	5	
Ovary	Normal	0	Yes
	Mild vascular congestion. No deform follicles	1	
	Moderate vascular congestion. Max. one deform follicle	2	
	Complete vascular congestion and serous exudates with fibrin spots. Deformed follicles	3	No
	Complete vascular congestion and fibrinopurulent exudate	4	
	Complete vascular congestion, fibrinopurulent exudate and deformed follicles	5	
Oviduct	Normal	0	Yes
	Vascular congestion	1	
	Vascular congestion, edema and serous exudates	2	
	Vascular congestion, edema and local fibrinopurulent exudates	3	No
	Vascular congestion, edema and diffuse fibrinopurulent exudates	4	
	Vascular congestion, edema, diffuse fibrinopurulent exudates and necrosis	5	

48 h after challenge all birds were euthanized and scoring of lesions was done accordingly. Scores 0, 1 and 2 were regarded as inflammatory reaction without infection and considered as a protective response against infection. Scores 3, 4 and 5 was regarded as inflammation with infection and considered as no protection of the response, i.e., infection had overwhelmed the inflammatory response.

### Collection of sera

Collection of sera for the ELISA was done before each immunization, before the challenge, and before euthanasia of birds included in the cross-protection study (experiment 2). Briefly, 3 mL blood was drawn aseptically from the brachial vein with cannula and syringe. All blood was stored at 5 °C until the next day. Sera were collected by centrifugation (10 min, 1800 × *g*), and stored at −20 °C until further use.

### ELISA

Wells in microtiter plates (Nunc-Immuno™ MicroWell™ 96-Well Plates, Thermo Scientific) were coated overnight at 4 °C with 0.5 µg recombinant protein (48 wells per protein) diluted in carbonate-bicarbonate buffer (pH 9.6) (Sigma-Aldrich). Each well was washed three times using 350 µL wash buffer (PBS + 0.05% Tween 20). The wells were blocked 2 h at room temperature with 200 µL blocking solution (PBS + 0.05% Tween 20 + 2% bovine serum albumin (BSA)) and washed once with wash buffer. Serum of birds from the same group was pooled for each time point yielding four pools of serum; GtxA-N, GtxA-C, FlfA, and placebo. The antibody titers from the vaccinated groups were obtained using serial threefold dilutions of chicken serum ranging from 1:300 to 1: 656 100 while serial twofold dilutions, ranging from 1:300 to 1:409 600 were used for the non-immunized group. In all plates, serum from the corresponding non-immunized group was included as control. All dilutions were prepared in triplicates in diluting buffer (PBS + 0.05% Tween 20 + 0.1% BSA), 100 µL of each dilution was added in triplicates and plates were incubated 1 h at 37 °C. For each protein, secondary antibody alone was used as a measure of background, while diluting buffer alone was used as a negative control. Following incubation, the wells were washed and 100 µL polyclonal goat anti-chicken IgG (Fc): HRP (AbD Serotec, diluted 1:4000 in diluting buffer) was added to each well. The plates were incubated 1 h at 37 °C and washed. To detect binding, 100 µL TMB substrate (Sigma) was added to each well. The plates were incubated 2 min, the reaction was stopped by adding 100 µL 1 M HCl and the absorbance was read at 450 nm by use of a PowerWave XS spectrophotometry (BioTek Instruments). Linear regression was done on the descending, linear section of the resulting sigmoid plot and the average background score plotted horizontally. The titer for each group was then found as the intersection between the background average and the linear regression line.

### Statistical analysis

All analysis of was done with SAS, version 9.3 (SAS Institute inc., Car, NC, USA). Lesion scores were compared using Fishers exact test. Bacteriology scores were compared using Wilcoxon Ranked test. Mean values of ELISA-titers were compared with one-sided *t* test. *P*-values below 0.05 were regarded as significant.

## Results

### Lesions

**Figure 1:**
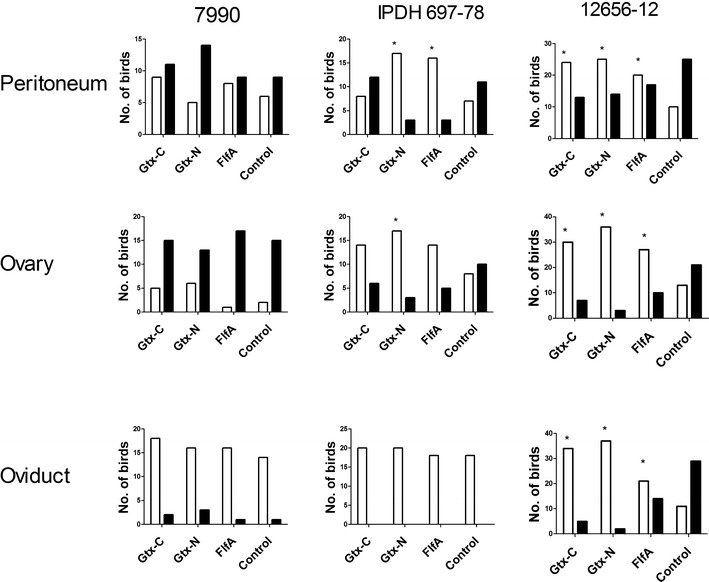
**Lesion scores in immunized or non-immunized birds 48** **h after challenge.** The columns show the number of birds with a protective response (white bars) or a non-protective response (black bars). Groups were immunized twice with GtxA-N, GtxA-C, FlfA or buffer only (placebo), prior to challenge. Significant differences (*P* < 0.05) to the control group are marked with *.

After 48 h of challenge, lesions found in all the challenged groups were local to diffuse peritonitis with varying amounts of purulent material. The exudates varied from serous, fibrinous clots to confluent, purulent material. Vascular engorgement of the ovary, oviduct and peritoneum was also found with purulent oophoritis and folliculitis involving one or more follicles. Regression and deformation of the ovarian follicles were found in some cases. Exsudative focal or diffuse salpingitis was found in few birds with serous or purulent material. Overall, strain 7990 caused the most severe lesions in the birds, which were mostly located in the peritoneum and the ovary. The birds infected with strain IPDH 697-78 did not show lesions in the oviduct regardless of immunization status. In regard of strain 12656-12, lesions were found in the peritoneum, ovary and oviduct. No lesions were found at the site of injection. The lesion scores are summarized in Figure 1.

The effect of immunization of the recombinant proteins differed depending on the challenge strain used. Immunization with any of the three proteins had no effect on the lesions scores when challenging with strain 7990 as compared to the non-immunized controls. In the groups challenged with strain IPDH 697-78, significantly lower lesions score in the peritoneum (*P* = 0.006) and the ovary (*P* = 0.02) was found after immunization with Gtx-N. Also immunization with FlfA caused lower lesion scores in the peritoneum (*P* = 0.006) when challenging with strain IPDH 697-78. Protection from the homologous strain 12656-12 was found when immunizing with Gtx-N (peritoneum: *P* = 0.03; ovary: *P* < 0.0001 and oviduct: *P* < 0.0001), Gtx-C (peritoneum: *P* = 0.02; ovary: *P* = 0.04; oviduct: *P* < 0.0001) and FlfA (peritoneum: *P* = 0.03; ovary: *P* = 0.03; oviduct: *P* = 0.04).

### Bacteriology

**Figure 2:**
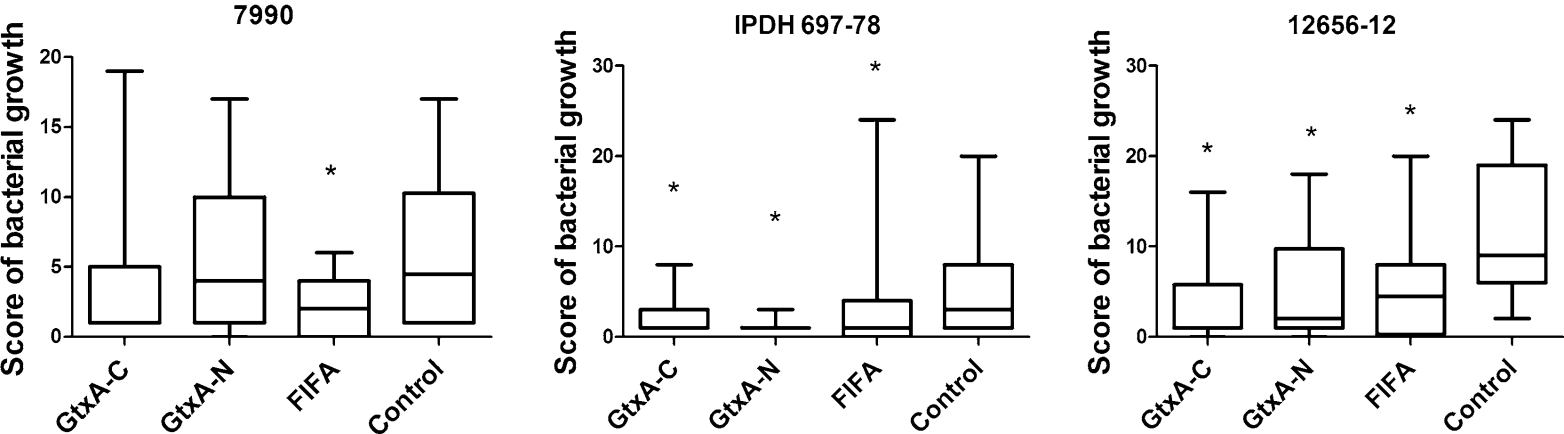
**Re-isolation of** ***G. anatis*** **in immunized or non-immunized birds 48** **h after challenge.** The growth of *G. anatis* was scored 0–5: 0 (no colonies), 1 (<10 colonies), 2 (10–200 colonies), 3 (>200 colonies), and 4 (dense/florid growth). The total bacterial load was calculated by summation of all scores. Significant differences (*P* < 0.05) to the control group are marked with *.

All the birds were positive for *G. anatis* at the initiation of the experiment. The isolation rates of *G. anatis* in pure culture from organs from the different groups are summarized in Figure 2. In the birds challenged with strain 7990, only immunization with FlfA resulted in lower bacterial isolation rates (*P* = 0.01). In contrast, significantly lower scores were found between the non-immunized and the immunized groups challenged with strain 12656-12 (*P*-values for GtxA-C: 0.0002, Gtx-N: 0.001, FlfA: 0.01) and with strain IPDH 697-78 (*P*-values for GtxA-C: 0.04, Gtx-N: 0.003, FlfA: 0.027).

### Antibody titers

**Figure 3:**
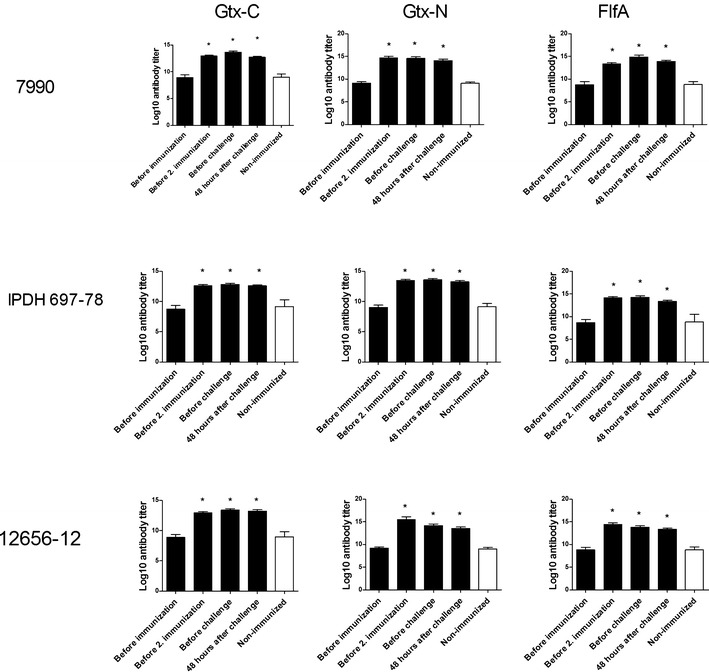
**Titer values of protein specific IgG in serum.** Sera was obtained and analyzed from immunized and non-immunized birds before each immunization, before challenge, and 48 h after challenge. Sera from non-immunized birds did not show any significant differences at the different time-points and are therefore represented with one bar. Significant differences (*P* < 0.05) to the control group are marked with *.

Birds immunized with any of the three recombinant proteins generated a protein-specific serological IgG response (Figure 3) as compared to the birds receiving buffer and adjuvant. This IgG level remained elevated compared to prior immunization for the whole immunization period in all the groups. However, after the challenge with *G. anatis*, all groups of birds showed a decrease in the protein-specific IgG level. No difference was observed in titer levels between groups challenged with the different strains of *G. anatis*, and no difference in titer levels was found between the sampling points for the non-immunized controls.

## Discussion

The aim of this study was to evaluate whether three recombinant proteins, previously described as major virulence factors of *G. anatis* [6] and potential immunogens [18, 20], could induce a cross-protective response against three different strains of *G. anatis*. Both FlfA and GtxA-N have previously been demonstrated to induce a protective immunity towards the homologous strain 12656-12 [18, 20], however, the cross-protective potential has not been investigated up until now. The results presented in this study showed that all three antigens induced a significant rise in protein-specific antibodies, as well as a protective immune response measured by lesion scores and bacterial re-isolation scores, against one or more of the three strains included in the challenge. However, none of the proteins were able to elicit full protection against all three bacterial strains. Relying on a single protein for cross-protective immunization therefore appears insufficient using the current protocol, and future studies investigating combinations of immunogens and/or additions of other immune enhancing compounds are needed.

*G. anatis* is a widespread opportunistic pathogen and constitutes part of the normal microbiota of the lower reproductive tract and the upper respiratory tract of egg-laying hens [1]. The hens used in the present study were all positive for *G. anatis* in the clocae. To avoid interference of this finding naïve or SPF-hens could be used. However, both alternatives have downsides to the outcome of the study. The dispersion of *G. anatis* among egglaying hens makes it difficult to find naïve hens [1]. SPF-hens is declared free of a list of pathogens, however, *G. anatis* is not included and to the authors knowledge therefore not known to be free from *G. anatis.* Furthermore, the intention of this vaccine is that it should be used in the broad population of egg-laying hens and therefore, a strong vaccine candidate should be able to to protect against infection under such circumstances.

The vast genetic diversity among *G. anatis* isolates has been described in several studies through analysis of genomic data [10, 22]. Additionally, in vivo studies have indicated that there is a difference in the lesion types found using different isolates of *G. anatis* [5]. This diversity in pathogenicity and virulence among different strains are also confirmed in this study. Challenge infections with the Mexican strain 7990 caused the highest lesions scores, which is in good agreement with previous findings showing that this isolate has a high in vivo virulence [5]. In contrast, strain IPDH 687-78 did not cause any lesions in the oviduct. Strain 12656-12 has previously been used in intraperitoneal challenge models and found to cause lesions in both the peritoneum, oviduct and ovary [8, 18, 20, 21]. These differences in the virulence and pathogenicity between different strains of *G. anatis* confirm the need for a broadly protective vaccine strategy and strongly suggest that ability to protect against heterologous challenge will be a critical limitation of successful vaccination against *G. anatis.*

Full in vivo protection against the homologous challenge was confirmed for GtxA-N and FlfA [20, 21], as well as for GtxA-C, for which the immunogenic abilities has only previously been tested in vitro [18]. A cross-protective potential of GtxA-N, defined by lower lesion scores in the peritoneum and the ovary and lower re-isolation rates, were observed when challenging with strain IPDH 697-78. As no lesions were found in the oviduct of the non-immunized birds challenged with this strain, GtxA-N can be described as giving full protection using this challenge model. The difference in immunogenic potential between the C- and N-terminal of GtxA has also been seen in the RTX-toxin produced by *P. multocida*, were the C-terminal has been found to provide the best protection [23]. Immunization with FlfA also showed cross-protective potential by lowering the lesion scores in peritoneum and, although non-significant, in the ovary after challenge with strain IPDH 697-78. Additionally, re-isolation scores from birds challenged with strain IPDH 697-78 also showed that all three proteins were able to reduce these.

While the above-mentioned results are encouraging, this study had limitations. Firstly, no protection was observed in the birds challenged with strain 7990, and secondly, high lesions scores were found in all groups challenged with strain 7990, although re-isolation of *G. anatis* was significantly lower for birds immunized with FlfA. As mentioned above, strain 7990 of *G. anatis* has previously been found to have a high in vivo virulence potential [5] and this could influence the results. To improve the level of protection, several possibilities are available, such as immunization with combined antigens [24] and/or alternative delivery methods [25].

The level of specific IgG found in serum shows that all three recombinant proteins used for immunization elicited an antigen-specific immune response following the initial immunization, despite the different outcomes of the challenges. This demonstrates that a high level of protein-specific antibodies does not automatically lead to protection. Likewise, a study using a reverse vaccinology approach used to identify antigens for a vaccine against *P. multocida* screened 71 proteins and found them all to be immunogenic, but only one protein actually induced protection following immunization [15].

All birds had low, yet detectable levels, of protein-specific antibodies prior to the initial immunization as seen in previous studies [18, 20]. This was not surprising as all birds cultured positive for *G. anatis* from the cloacal swabs. However, as all three *G. anatis* strains caused infection in the non-immunized birds in the control groups, this background level of antibodies was not adequate to protect against challenge. Thus, a mucosal presence of resident strains of *G. anatis* does not appear to induce a protective immune response.

Based on the present investigation, we conclude that at least two of the tested vaccine prototypes, GtxA-N and FlfA, elicit a promising, cross-protective immune response and therefore have a considerable potential for the development of a serotype-independent vaccine against *G. anatis.* To provide a higher degree of serotype-independent protection, future investigations should aim at identifying efficient combinations of immunogens with immune-stimulatory additions. Furthermore, the effect of vaccination on production performance indicators including morbidity, mortality and egg-laying should be investigated.
